# Cognitive Dysfunction and Malnutrition Are Independent Predictor of Dysphagia in Patients with Acute Exacerbation of Congestive Heart Failure

**DOI:** 10.1371/journal.pone.0167326

**Published:** 2016-11-29

**Authors:** Junichi Yokota, Yoshiko Ogawa, Shinsuke Yamanaka, Yoshimi Takahashi, Hiroshi Fujita, Nobuhiro Yamaguchi, Noriko Onoue, Takeshi Ishizuka, Tsuyoshi Shinozaki, Masahiro Kohzuki

**Affiliations:** 1 Department of Internal Medicine and Rehabilitation Science, Tohoku University Graduate School of Medicine, Sendai, Japan; 2 Department of Rehabilitation, Sendai Medical Center, Sendai, Japan; 3 Department of Sport and Medical Sciences, Faculty of Medical Technology, Teikyo University, Tokyo, Japan; 4 Department of Cardiology, Sendai Medical Center, Sendai, Japan; West Virginia University School of Medicine, UNITED STATES

## Abstract

Early detection and intervention for dysphagia is important in patients with congestive heart failure (CHF). However, previous studies have focused on how many patients with dysphagia develop CHF. Studies focusing on the comorbidity of dysphagia in patients with CHF are rare. Additionally, risk factors for dysphagia in patients with CHF are unclear. Thus, the aim of this study was to clarify risk factors for dysphagia in patients with acute exacerbation of CHF. A total of 105 patients, who were admitted with acute exacerbation of CHF, were enrolled. Clinical interviews, blood chemistry analysis, electrocardiography, echocardiography, Mini-Mental State Examination (MMSE), exercise tolerance tests, phonatory function tests, and evaluation of activities of daily living (ADL) and nutrition were conducted on admission. After attending physicians permitted the drinking of water, swallowing screening tests were performed. Patients were divided into a dysphagia group (DG) or a non-dysphagia group (non-DG) based on Functional Oral Intake Scale level. Among the 105 patients, 38 had dysphagia. A greater number of patients had history of aspiration pneumonia and dementia, and there was a higher age, N-terminal pro-B-type natriuretic peptide level in the DG compared with the non-DG. MMSE scores, exercise tolerance, phonatory function, status of ADL, nutrition, albumin, and transthyretin were lower in the DG compared with the non-DG. In multivariate analysis, after adjusting for age and sex, MMSE, BI score, and transthyretin was independently associated with dysphagia. Comorbidity of dysphagia was 36.1% in patients with acute exacerbation of CHF, and cognitive dysfunction and malnutrition may be an independent predictor of dysphagia.

## Introduction

Heart failure is a global public health problem. In the United States alone, the prevalence is 5.7 million, with 870,000 new cases diagnosed each year [1]. Similarly, in Japan, the increasing number of patients with heart failure is concerning. Indeed, the number of Japanese outpatients with left ventricular dysfunction was 979,000 in 2005 [2]. It is projected that the incidence of left ventricular dysfunction will first rapidly and then gradually increase to a peak of 1.32 million patients by 2035, with a rapid acceleration occurring by 2020 [2]. Accordingly, congestive heart failure (CHF) patients having various comorbidity and medical history has been increasing. In particular, a various comorbidity occurs in patients with acute exacerbation of CHF various comorbidity and one is a dysphagia.

Little is known about relationship between CHF and dysphagia, however, some investigation results has been reported. Respiratory diseases, chronic obstructive pulmonary disease, and xerostomia, were indicated as risk factors for oropharyngeal dysphagia [3]. Another retrospective study reported a high incidence of risk for oropharyngeal dysphagia in hospitalized patients, and hospitalized patients with CHF. Moreover, dysphagia increases length of hospital stay 1.8 times in patients with CHF [4]. Moreover, Zhu, et al. have shown that high blood pressure and heart failure are important risk factors for hospital-acquired pneumonia in cardiovascular inpatients, and that contracting hospital-acquired pneumonia significantly increases morbidity, mortality, hospitalization stays, and total medical costs [5]. It is suspected that CHF is strongly related to dysphagia.

Dysphagia is a risk factor for malnutrition and incidence of lower respiratory tract infection in the independently-living elderly population [6]. In addition, a recent investigation demonstrated that dysphagia was related to sarcopenia or the causes of sarcopenia in hospitalized elderly patients without a history of treatment for stroke, and without diagnosis of neurodegenerative disease [7]. CHF patients with cardiac cachexia, caused by malnutrition, have mortality 2–3 times higher compared with non-cachectic CHF patients [8]. Therefore, dysphagia may affect prognosis in patients with CHF through malnutrition, sarcopenia, and cardiac cachexia, as well as aspiration pneumonia.

However, previous studies have focused on how many patients with dysphagia develop CHF. Prospective studies focusing on the comorbidity of dysphagia in patients with CHF are rare. Therefore, ratio of comorbidity of dysphagia and risk factors for dysphagia in patients with CHF are unclear. Since aspiration pneumonia and malnutrition may affect clinical condition and prognosis, early detection and intervention for dysphagia is important in patients with acute exacerbation of CHF. Thus, the aim of the present study was to clarify the comorbidity of dysphagia and risk factors in patients with acute exacerbation of CHF.

## Materials and Methods

### Subjects and Study Design

In this study, 150 consecutive patients, who were admitted to the Department of Cardiology, National Hospital Organization Sendai Medical Center, with acute exacerbation of CHF from May 2015 to May 2016, were admitted. Patients, who were under 20 years of age and were judged inappropriate for the present study, were excluded. Of the 150 patients admitted, 12 were judged inappropriate because of severe mental disease in five patients, agitation in four patients, and severe depression in the remaining patient. A total of 33 patients did not give consent to participate and were therefore excluded. Therefore, a total of 105 patients were registered.

**Fig 1:**
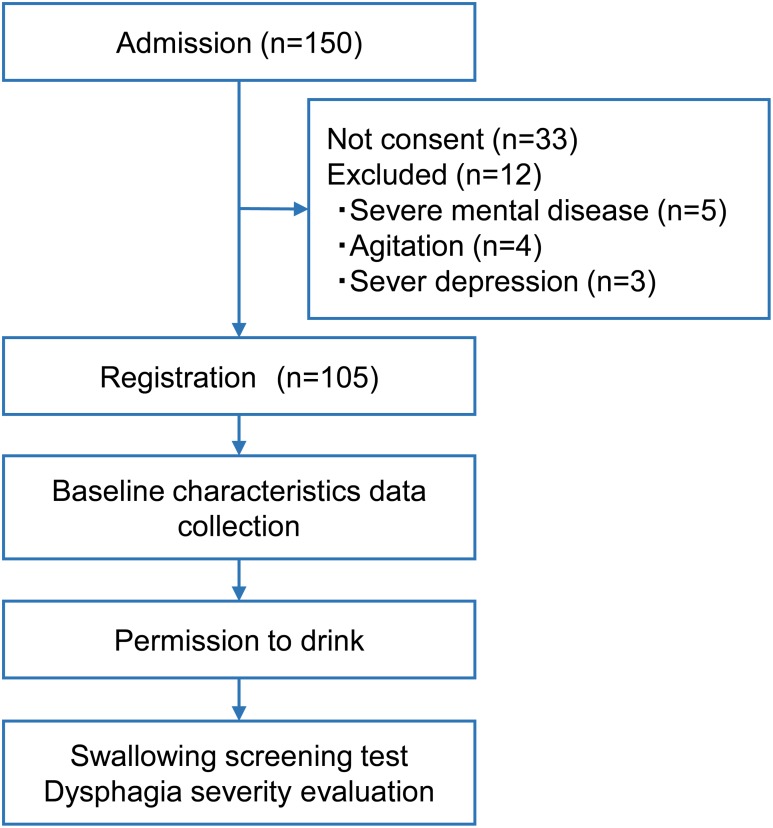
Study design. Baseline characteristics of the patients was collected after registration. Swallowing screening tests were performed after attending physicians permitted the drinking of water and dysphagia severity were evaluated.

This study used a cross-sectional design, as shown in Fig 1. Information concerning baseline characteristics of the patients was collected after registration. Swallowing screening tests were performed after attending physicians permitted the drinking of water.

This study was approved by the Ethics Committee of Tohoku University Graduate School of Medicine (Approval No. 2015-1-060), and the Ethics Committee of Sendai Medical Center (Approval No. 27–1). All participating patients provided written informed consent.

### Baseline Characteristics and Data Collection

Clinical information on admission, including age, sex, height, weight, body mass index (BMI), medical history, complications of diabetes, hypertension, and dyslipidemia, medications using angiotensin converting enzyme inhibitor, angiotensin receptor blocker, statin, calcium antagonists, diuretics, beta-blocker, and digitalis, smoking history, and alcohol history, was obtained from interviews with the patients or family members. Prehospital Functional Oral Intake Scale (FOIS) [9] score was also obtained. The FOIS is a 7-point scale and measures the level of independence of functional oral intake (Table 1). Levels 1–3 relate to varying degrees of non-oral feeding, and levels 4–7 relate to varying degrees of oral feeding without non-oral supplementation. These latter scale levels consider both diet modifications and patient compensations [9]. Clinically significant dysphagia was defined as requirement for modification of an oral diet (FOIS grades 1–5) [7, 10]. According to previous studies, dysphagia was defined as having a FOIS level of 5 or less in the present study [7, 10].

**Table 1:** Functional Oral Intake Scale [9].

Level 1	Nothing by mouth.
Level 2	Tube dependent with minimal attempts of food or liquid.
Level 3	Tube dependent with consistent oral intake of food or liquid.
Level 4	Total oral diet of a single consistency.
Level 5	Total oral diet with multiple consistencies, but requiring special preparation or compensations.
Level 6	Total oral diet with multiple consistencies without special preparation, but with specific food limitations.
Level 7	Total oral diet with no restrictions.

Diagnosis of New York Heart Association (NYHA) functional class was determined by cardiologists. Blood chemistry analysis, electrocardiography, and echocardiography were performed.

Cognitive function was evaluated using the Mini-Mental State Examination (MMSE) [11]. Exercise tolerance was assessed using handgrip strength [12] and the Modified Specific Activity Scale (MSAS) [13], which is a modification of the Specific Activity Scale [14], to improve sensitivity and specificity. Phonatory function was evaluated using Maximum Phonation Time (MPT) data [15]. Activities of daily living (ADL) were evaluated using the Barthel Index (BI) [16]. Prehospital BI and prehospital MSAS scores were also evaluated. Arm circumference (AC), triceps skin fold (TSF), arm muscle circumference (AMC), arm muscle area (AMA) [17], and controlling nutritional status (CONUT) [18] were evaluated as a measurement of nutritional status. Anthropometric data were standardized according to age- and gender-stratified Japanese anthropometric reference data and expressed as percent arm circumference (%AC), percent TSF (%TSF), percent AMC (%AMC), and percent AMA (%AMA) [19]. CONUT, a nutritional assessment tool that assesses protein metabolism, lipid metabolism, and immune function using three parameters (serum albumin, total cholesterol, and total lymphocyte count), was calculated [18].

### Swallowing Screening Test

The Repetitive Saliva Swallowing Test (RSST) [20, 21] and the Water Swallowing Test (WST) [22] were performed.

RSST, in which swallowing movements can be identified by inspection and palpation of the prominentia laryngea, enables assessment of the potential to swallow saliva; it is performed by counting the frequency of swallows over 30 seconds. When the number of dry swallowings was less than three times during 30 seconds, swallowing function of the patient was estimated as abnormal.

The WST was performed in the following procedures. Cold water (3 ml) was placed on the floor of the mouth. The patient was then instructed to swallow. If the patient was unable to swallow, or experienced dyspnea, coughing, or wet-hoarse dysphonia after swallowing, the test was finished and a score was judged (1 for inability to swallow, 2 for dyspnea, and 3 for cough or dysphonia). Otherwise, the patient was asked to perform two dry swallows. If the patient was unable to perform either of the two dry swallows, a score of 4 was judged. If the patient was both dry swallows, a score of 5 was judged. The entire procedure was repeated twice more. Swallowing function of the patient was estimated as abnormal when the score was less than 4.

### Data and Statistical Analysis

Patients were divided into two groups: dysphagia group (DG) and non-dysphagia group (non-DG). All data are expressed as mean ± standard deviation (SD) for continuous variables, and counts and percentages for categorical variables. Comparisons between groups were made using unpaired two tailed t tests for continuous variables and Chi-square tests for categorical variables. Predictors of dysphagia were assessed using logistic regression analysis. A univariate analysis was performed, and significant variables thought to be involved in comorbidity of dysphagia were entered into a multivariate model. In the logistic regression analysis, multicollinearity was assessed using the variance inflation factor (VIF) [23]. VIF values exceeding 10 are regarded as indicating serious multicollinearity [23].

Predictors were expressed as odds ratios (OR) with 95% confidence intervals (CI). All analyses were performed using SPSS 21.0 for Windows (SPSS Inc. Chicago, IL). Statistical significance was accepted at P<0.05.

## Results

### The Comorbidity of Dysphagia

Among the 105 patients, dysphagia was observed in 38 patients. The mean FOIS level in the DG was significantly lower compared with the non-DG (3.3 ± 1.1 vs. 6.8 ± 0.4 P<0.001). The DG also had significantly lower prehospital FOIS compared with the non-DG (6.2 ± 0.9 vs. 6.8 ± 0.4 P<0.001). The number of patients receiving parenteral nutrition and enteral nutrition was 23 (60.5%) in the DG.

### Baseline Characteristics

Demographic characteristics is shown in Table 2. Age was significantly higher in the DG compared with the non-DG. There were no significant difference in the sex, height, weight, and BMI between the groups. In the DG, the number of patients with history of smoking abuse was significantly lower compared with the non-DG. The number of patients who had a history of aspiration pneumonia and dementia was significantly higher in the DG compared with the non-DG. Furthermore, there were more denture-wearers in the DG compared with the non-DG.

**Table 2:** Demographic characteristics.

	DG(n = 38)	non-DG(n = 67)	P-value
Age (years)	82.7±8.8	75.9±10.8	0.001
Sex (male/female)	18/20	37/30	0.439
Height (cm)	154.0±10.5	157.1±9.7	0.130
Weight (kg)	53.8±13.7	57.9±13.3	0.141
BMI (kg/m^2^)	22.5±4.5	23.4±4.4	0.361
Medical history			
Angina pectoris	14 (36.8)	33 (49.3)	0.219
Myocardial infarction	10 (26.3)	17 (25.4)	0.915
Valvular disease	13 (34.2)	20 (29.9)	0.644
Cerebrovascular disease	11 (28.9)	21 (31.3)	0.798
Neuromuscular disease	2 (5.3)	1 (1.5)	0.296
Respiratory disease	4 (10.5)	10 (14.9)	0.524
Aspiration pneumonia	11 (28.9)	2 (3.0)	<0.001
Cancer	12 (31.6)	12 (17.9)	0.109
Dementia	9 (23.7)	2 (3.0)	0.002
Mental illness	4 (10.5)	7 (10.4)	0.616
Complication			
Diabetes	14 (36.8)	29 (43.3)	0.519
Hypertension	29 (76.3)	46 (68.7)	0.404
Dyslipidemia	11 (28.9)	26 (38.8)	0.310
Medication			
ACEI	7 (18.4)	24 (35.8)	0.060
ARB	12 (31.6)	26 (38.8)	0.459
Statin	9 (23.7)	16 (23.9)	0.982
Calcium antagonists	15 (39.5)	25 (37.3)	0.827
Diuretics	33 (86.8)	61 (91.0)	0.358
Beta-blockers	7 (18.4)	24 (35.8)	0.060
Digitalis	3 (7.9)	10 (14.9)	0.233
Smoking	11 (28.9)	37 (55.2)	0.009
Alcohol	14 (36.8)	35 (52.2)	0.129
Use of dentures	31 (81.6)	40 (59.7)	0.021

Values are means ± standard deviation or numbers of subjects per group (n) with percentages.

DG, Dysphagia Group; non-DG, non-Dysphagia Group; BMI, body Mass Index; ACEI, Angiotensin Converting Enzyme Inhibitor; ARB, Angiotensin Receptor Blocker.

Table 3 shows the results of NYHA functional class, echocardiography, electrocardiography, and blood chemistry analyses. NYHA functional class was significantly higher in the DG compared with the non-DG. Furthermore, the DG had significantly higher N-terminal pro-B-type natriuretic peptide (NT-proBNP), blood urea nitrogen (BUN), and C-reactive protein (CRP) levels compared with the non-DG, and significantly lower hemoglobin, hematocrit, total lymphocyte count (TLC), albumin, and transthyretin compared with the non-DG. However, there was no significant difference in ejection fraction between the groups. In this study, the patients diagnosed heart failure with preserved ejection fraction (EF≧50%) were about 50% (21 patients in dysphagia group (55.3%) and 32 patients in non-dysphagia group (47.8%))

**Table 3:** Clinical and laboratory findings.

	DG(n = 38)	non-DG(n = 67)	P-value
NYHA class (I/II/III/IV)	0 / 10 / 18 / 10	6 / 19 / 37 / 5	0.012
Echocardiography			
Ejection fraction (%)	51.3±17.6	47.3±17.8	0.275
LVDd/BSA (mm/m^2^)	34.9±4.8	34.0±6.0	0.420
LVDs/BSA (mm/m^2^)	25.7±6.5	25.8±7.0	0.942
Electrocardiography			
Atrial fibrillation	22 (57.9)	36 (53.7)	0.680
Blood chemistry analysis			
NT-proBNP (pg/mL)	16,049.6±16,734.0	7,156.4±9,751.0	0.004
Hemoglobin (g/dL)	10.2±2.0	11.9±2.4	<0.016
Hematocrit (%)	31.3±5.8	36.1±6.8	<0.013
TLC (/μL)	1,131.1±667.1	1,404.1±668.5	0.015
BUN (g/dL)	33.0±18.7	25.2±13.5	0.016
Creatinine (g/dL)	1.4±1.0	1.2±0.8	0.119
Urinary acid (mg/dL)	7.0±2.3	6.9±2.3	0.778
eGFR (mL/min/1.73m^2^)	47.1±29.0	57.0±27.5	0.087
CRP (mg/dL)	3.7±5.2	1.6±3.5	0.015
Total Protein (g/dL)	6.4±0.7	6.5±0.6	0.283
Albumin (g/dL)	3.4±0.5	3.6±0.5	0.032
Transthyretin (mg/dL)	15.1±4.3	17.6±5.0	0.012
RBP (mg/dL)	2.6±1.0	2.8±1.3	0.590
Transferrin (mg/dL)	216.8±63.2	230.9±56.2	0.240
Total cholesterol (g/dL)	169.1±46.2	160.0±34.8	0.262
HDL cholesterol (g/dL)	49.8±13.2	48.9±13.4	0.738
LDL cholesterol (g/dL)	97.4±44.3	91.6±28.7	0.419
Triglyceride (g/dL)	78.9±33.9	83.0±33.8	0.557
HbA1c (%)	6.1±0.8	6.4±1.2	0.191

Values are means ± standard deviation or numbers of subjects per group (n) with percentages.

DG, Dysphagia Group; non-DG, non-Dysphagia Group; NYHA, New York Heart Association; BSA, Body Surface Area; LVDd, Left Ventricular end-diastolic Diameter; LVDs, Left Ventricular end-systolic Diameter; NT-proBNP, N-Terminal pro-B-type Natriuretic Peptide; TLC, Total Lymphocyte Count; BUN, Blood Urea Nitrogen; CRP, C-Reactive Protein; GFR, estimated Glomerular Filtration Rate; HDL, High Density lipoprotein; LDL, Low Density Lipoprotein; RBP, Retinol Binding Protein.

Results of the cognitive function test, exercise tolerance tests, phonatory function test, and evaluation of status of ADL and nutrition are shown in Table 4. There was no significant difference in the mean number of days for evaluation between the groups. MMSE scores were significantly lower in the DG compared with the non-DG. The DG had significantly lower handgrip strength, MSAS, and BI compared with the non-DG. The DG also had significantly lower prehospital MSAS and prehospital BI compared with the non-DG. The DG had significantly lower MPT compared with the non-DG. However, there were no significance difference in the nutritional status between two groups.

**Table 4:** Cognitive function, physical performance and nutritional status.

	DG(n = 38)	non-DG(n = 67)	P-value
Performed evaluation (day)	3.1±2.1	3.0±1.6	0.832
MMSE (score)	18.3±5.4	24.2±5.3	<0.001
Handgrip strength (kg)	14.9±6.8	22.5±8.9	<0.001
Prehospital MSAS (METs)	2.8±1.1	3.9±1.6	<0.001
MSAS (METs)	1.8±0.4	2.5±1.4	<0.001
MPT (second)	8.1±3.7*	14.1±7.6	<0.001
Prehospital BI (score)	71.8±29.0	90.5±17.5	0.001
BI (score)	23.4±22.0	59.3±28.0	<0.001
%AC (%)	98.8±14.2	101.5±14.1	0.346
%TSF (%)	95.4±58.4	105.9±49.8	0.329
%AMA (%)	102.4±22.1	107.5±27.4	0.328
%AMC (%)	100.4±10.8	102.8±12.6	0.318
CONUT (score)	4.3±3.2	3.3±2.5	0.070

Values are means ± standard deviation.

*Calculated only for those who performed MPT (n = 37 in the DG and n = 67 in the non-DG).

DG, Dysphagia Group; non-DG, non-Dysphagia Group; MMSE, Mini-Mental State Examination; MSAS, Modified Specific Activity Scale; MPT, Maximum Phonation Time; BI, Barthel Index; AC, Arm Circumference; TSF, Triceps Skin Fold; AMA, Arm Muscle Area; AMC, Arm Muscle Circumference; CONUT, Controlling Nutritional Status.

### Swallowing Function

Table 5 presents the results of the swallowing screening tests. There were no significant difference in the mean number of days for evaluation and until patients were permitted the drinking after hospitalization between the groups. However, the mean number of days until patients was able to eat after hospitalization in the DG was significantly greater compared with the non-DG. The number of patients who were positive for the RSST was significantly higher in the DG compared with the non-DG. The DG had significantly lower WST scores compared with the non-DG. No patients were using sedative or hypnotic medication during evaluations.

**Table 5:** Swallowing screening tests.

	DG(n = 38)	non-DG(n = 67)	P-value
Performed evaluation (day)	3.1±2.1	2.9±1.6	0.622
Drinking start (day)	1.2±0.4	1.3±0.9	0.530
Diet start (day)	3.0±3.1	1.5±1.0	<0.001
RSST positive	25 (65.8)	10 (14.9)	<0.001
WST (score)	3.2±0.7	4.4±0.9	<0.001

Values are means ± standard deviation or numbers of subjects per group (n) with percentages.

DG, Dysphagia Group; non-DG, non-Dysphagia Group; RSST, Repetitive Saliva Swallowing Test; WST, Water Swallowing Test.

### Predictors of Dysphagia

Results from logistic regression analysis of risk factors for dysphagia are shown in Table 6. Analysis was performed adjusting for age and sex. Variables whose VIF values exceeded 10 were hemoglobin and hematocrit; therefore, hemoglobin and hematocrit were excluded from the multivariate model, according to the bibliography [23]. Variables such as medical history of aspiration pneumonia and dementia, use of dentures, NT-proBNP, TLC, BUN, CRP, albumin, transthyretin, MMSE, handgrip strength, prehospital MSAS and MSAS, MPT, and prehospital BI and BI, were entered into a multivariate model. There were significance difference in the MMSE (OR = 1.155, 95% CI: 1.020–1.306, P = 0.023), transthyretin (OR = 1.136, 95% CI: 1.002–1.287, P = 0.046) and, BI (OR = 1.043, 95% CI: 1.016–1.070, P = 0.001) after adjusting for age and sex.

**Table 6:** Predictors of dysphagia.

	β	SE	Wald	P-value	OR	95%CI
MMSE	0.144	0.063	5.200	0.023	1.155	1.020–1.306
Transthyretin	0.127	0.210	8.732	0.046	1.136	1.002–1.287
BI	0.042	0.013	10.138	0.001	1.043	1.016–1.070

Analysis was performed adjusting for age and sex.

MMSE, Mini-Mental State Examination; BI, Barthel Index; SE, Standard Error; OR, Odds Ratio; CI, Confidence Interval.

## Discussion

This prospective study showed the comorbidity of dysphagia in patients with acute exacerbation of CHF. Dysphagia was observed in 36.1% of patients. Comorbidity of dysphagia was previously shown to be 50.4% with acute stroke [24], 81.1% with Parkinson’s disease [25], and 20.5% in COPD of acute exacerbation [26], which frequently causes dysphagia. The incidence of comorbidity of dysphagia in patients with acute exacerbation of CHF was lower than acute stroke patients, however, higher than patients with acute exacerbation of COPD. Moreover, the incidence of comorbidity with dysphagia in patients with CHF in the present study is similar to comorbidity rates with metabolic syndrome and chronic kidney disease in patients with CHF (both approximately 40%) [27], but higher than other comorbidities in patients with CHF such as anemia (approximately 30%), stroke (approximately 20%), and cancer (approximately 10%) [27].

MMSE score was found to be an independent predictor of dysphagia in patients with acute exacerbation of CHF after adjusting for age and sex. Prevalence of swallowing difficulties in patients with dementia ranged from 13 to 57% in a previous meta-analysis [28]. In the previous study, the alzheimer disease (AD) patients were impaired in “oral transit delay” with liquids, whereas the vascular dementia (VaD) patients showed deficits in ‘‘bolus formation and mastication” of semisolid food, ‘‘hyolaryngeal excursion”, ‘‘epiglottic inversion”, and ‘‘silent aspiration” [29]. Moreover, “delayed swallowing reflex” was observed both of patients [29]. Thus, the previous study reported that the comorbidity of dysphagia is high in patients with dementia, and the swallowing disorders of the AD may result from sensory impairment in relation to dysfunctions in the temporoparietal areas, whereas the swallowing disorders of VaD may primarily be caused by motor impairments due to disruptions in the corticobulbar tract [29].

According to another meta-analysis, the prevalence of dementia in individuals aged over 60 was 5–7% [30]. However, cognitive dysfunction was observed in 52.6% of patients with CHF and cognitive function was associated with age and ejection fraction [31]. Chronic cerebral hypoperfusion [32] and acceleration of cerebral aging [33], caused by cardiac dysfunction [32], were factors involved in cognitive dysfunction in patients with CHF. Increased heart failure severity, longer disease duration, and increased B-type natriuretic peptide levels are associated with lower cerebral blood flow in patients with CHF [34]. Moreover, high levels of NT-proBNP are associated with increased risk of dementia in the elderly [35]. Thus, NT-proBNP, BNP, heart failure severity is risk factor of cognitive dysfunction in patients with CHF. Actually, in the present study, the DG had higher NT-proBNP levels and heart failure severity on admission compared with the non-DG. Therefore, NT-proBNP level and heart failure severity may be related to lower MMSE score in patients with dysphagia. All eight patients with a history of dementia were in the DG. However, 50% of patients had MMSE scores less than cut-off values (<24 points; 88.9% in the DG vs. 25.6% in the non-DG). Thus, the number of patients suspected of having cognitive dysfunction was higher in the DG compared with the non-DG, after adjusting for age and sex.

Moreover, transthyretin and BI were also independent predictor of dysphagia in patients with acute exacerbation of CHF. Albumin and transthyretin were significantly lower and CONUT tended to be higher in the DG, compared with the non-DG. These results show that the DG were malnutrition compared with the non-DG in admission. The DG might be malnutrition before admission, and it may have been caused by dysphagia and/or cardiac cachexia. Previous study reported that malnutrition impairs respiratory muscles [36] and skeletal muscles [37], and may cause swallowing muscle dysfunction [38, 39]. Additionally, it is shown that muscle weakness is an independent predictor of pharyngeal dysfunction [40]. In this study, the DG had significantly lower muscle strength and MWST score, and had significantly higher ratio of RSST positive, compared with the non-DG. This results suggested that the DG had pharyngeal dysfunction due to malnutrition and/or muscle strength. Furthermore, the DG had significantly lower BI and MSAS, and had significantly lower prehospital BI and MSAS, compared with the non-DG. These results suggest that patients with dysphagia had lower ADL and exercise tolerance before admission. Lower BI, malnutrition, and dysphagia were significantly associated with the incidence of aspiration pneumonia [41], and patients with lower ADL score had higher aspiration risk [42]. Therefore, it is necessary to be careful about drawing conclusions concerning the comorbidity of dysphagia in patients with CHF who had cognitive dysfunction, low levels of ADL, and malnutrition.

MPT was significantly lower in the DG compared with the non-DG. MPT is related to the severity of respiratory impairment [43]. Thus, lower MPT in patients with dysphagia may reflect respiratory muscle weakness due to malnutrition and/or generalized muscle weakness, and respiratory disorder due to acute exacerbation of CHF.

Additionally, patients who had a history of aspiration pneumonia were significantly higher in the DG; these patients may have had swallowing dysfunction before admission. Thus, it is important that comorbidity of dysphagia is investigated in patients with a medical history of aspiration pneumonia.

There are several limitations of the current study. First, the sample size was small. Second, videofluoroscopic or videoendoscopic swallowing examinations were not performed. Therefore, details concerning swallowing dysfunction remain unclear. Videofluoroscopic or videoendoscopic swallowing examinations are important for diagnosis of dysphagia. However, these examinations are invasive evaluation methods and cannot always be performed depending on conditions of patients, especially in acute-phase patients. In contrast, FOIS measured the level of independence of functional oral intake is a noninvasive evaluation method of dysphagia. Thus, FOIS is useful a useful method for evaluating dysphagia in acute-phase patients with CHF. Third, prognosis in patients with dysphagia and the effect of rehabilitation remain unclear. Further studies, such as a multicenter trial, longitudinal study, and intervention studies, are needed to clarify the relationship between dysphagia and CHF. Specifically, dysphagia caused by disuse syndrome due to heart failure severity and/or disease duration may respond to cardiac rehabilitation to improve dysphagia, as well as exercise tolerance and ADL.

### Conclusions

The present study revealed that comorbidity of dysphagia was high in patients with acute exacerbation of CHF. Additionally, MMSE, high levels of transthyretin, and BI were independent predictors of dysphagia. The results of the present study may aid early detection of patients with dysphagia, and may contribute to prevention of aspiration pneumonia, shortening length hospitalization, and improvement of prognosis in patients with acute exacerbation of CHF.

## References

[1] Mozaffarian D, Benjamin EJ, Go AS, Arnett DK, Blaha MJ, Cushman M, et al Heart disease and stroke statistics—2015 update: a report from the American Heart Association. Circulation 2014; 12; 131: e29–322, accessed March 9, 2016. 10.1161/CIR.0000000000000152 25520374

[2] Okura Y, Ramadan MM, Ohno Y, Mitsuma W, Tanaka K, Ito M, et al Impending epidemic: future projection of heart failure in Japan to the year 2055. Circ J 2008; 72: 489–491. 18296852 10.1253/circj.72.489

[3] Bassi D, Furkim AM, Silva CA, Coelho MS, Rolim MR, Alencar ML, et al Identification of risk groups for oropharyngeal dysphagia in hospitalized patients in a university hospital. Codas 2014; 26: 17–27. 24714855

[4] Altman KW, Yu GP, Schaefer SD. Consequence of dysphagia in the hospitalized patient: impact on prognosis and hospital resources. Arch Otolaryngol Head Neck Surg 2010; 136: 784–789. 10.1001/archoto.2010.129 20713754

[5] Zhu J, Zhang X, Shi G, Yi K, Tan X. Atrial Fibrillation Is an Independent Risk Factor for Hospital-Acquired Pneumonia. PLoS One 2015; 10: e0131782, eCollection 2015. accessed March 9, 2016. 10.1371/journal.pone.0131782 26204447 PMC4512692

[6] Serra-Prat M, Palomera M, Gomez C, Sar-Shalom D, Saiz A, Montoya JG, et al Oropharyngeal dysphagia as a risk factor for malnutrition and lower respiratory tract infection in independently living older persons: a population-based prospective study. Age Ageing 2012; 41: 376–381. 22311895 10.1093/ageing/afs006

[7] Maeda K, Akagi J. Decreased tongue pressure is associated with sarcopenia and sarcopenic dysphagia in the elderly. Dysphagia 2015; 30: 80–87. 10.1007/s00455-014-9577-y 25248988

[8] Anker SD, Laviano A, Filippatos G, John M, Paccagnella A, Ponikowski P, et al ESPEN Guidelines on Parenteral Nutrition: on cardiology and pneumology. Clin Nutr 2009; 28: 455–460. 10.1016/j.clnu.2009.04.023 19515464

[9] Crary MA, Mann GD, Groher ME. Initial psychometric assessment of a functional oral intake scale for dysphagia in stroke patients. Arch Phys Med Rehabil 2005; 86: 1516–1520. 10.1016/j.apmr.2004.11.049 16084801

[10] Kim Y, Park GY, Seo YJ, Im S. Effect of anterior cervical osteophyte in poststroke dysphagia: a case-control study. Arch Phys Med Rehabil 2015; 96: 1269–1276. 10.1016/j.apmr.2015.02.026 25769673

[11] Folstein MF, Folstein SE, McHigh PR. ‘‘Mini-mental state”: a practical method of grading the cognitive function of patients for the clinician. J Psychiatr Res 1978; 12: 189–198. 10.1016/0022-3956(75)90026-6 1202204

[12] Izawa K, Hirano Y, Yamada S, Oka K, Omiya K, Iijima S. Improvement in Physiological Outcomes and Health-Related Quality of Life Following Cardiac Rehabilitation in Patients With Acute Myocardial Infarction. Cir J 2004; 68: 315–320. 10.1253/circj.68.315 15056827

[13] Adachi Y, Konishi H, Fukui T, Noguchi T, Kawakami R, Nakanishi M, et al An Examination of Validity of modified Specific Activity Scale. JJCR 2009; 14: 115–118. (in Japanese)

[14] Goldman L, Hashimoto B, Cook EF, Loscalzo A. Comparative reproducibility and validity of systems for assessing cardiovascular functional class: advantages of a new specific activity scale. Circulation 1981; 64: 1227–1234. 7296795 10.1161/01.cir.64.6.1227

[15] Speyer R, Bogaardt HC, Passos VL, Roodenburg NP, Zumach A, Heijnen MA, et al Maximum phonation time: variability and reliability. J Voice 2010; 24: 281–284. 10.1016/j.jvoice.2008.10.004 19111437

[16] Mahoney FI, Barthel DW. Functional Evaluation: The Barthel Index. Maryland State Med J 1965; 14: 61–65. 14258950

[17] Hosoya N, Okada T, Muto Y, Yamamori H, Tashiro T, Miwa Y, et al Japanese anthropometric reference data 2001 (JARD 2001). Japanese Journal of Nutritional Assessment. Med Rev 2002; 19 (Suppl): 1–81. (in Japanese)

[18] Ignacio de Ulíbarri J, González-Madroño A, de Villar NG, González P, González B, Mancha A, et al CONUT: a tool for controlling nutritional status. First validation in a hospital population. Nutr Hosp 2005; 20: 38–45. 15762418

[19] Wakahara T, Shiraki M, Murase K, Fukushima H, Matsuura K, Fukao A, et al Nutritional screening with Subjective Global Assessment predicts hospital stay in patients with digestive diseases. Nutrition 2007; 23: 634–639. 10.1016/j.nut.2007.06.005 17629455

[20] Oguchi K, Saitoh E, Mizuno M, Baba M, Okui M, Suzuki M. The repetitive saliva swallowing test (RSST) as a screening test of functional dysphagia (1) Normal values of RSST. Jpn J Rehabil Med 2000; 37: 375–382. (in Japanese)

[21] Oguchi K, Saitoh E, Baba M, Kusudo S, Tanaka T, Onogi K. The repetitive saliva swallowing test (RSST) as a screening test of functional dysphagia (2) Validity of RSST. Jpn J Rehabil Med 2000; 37: 383–388. (in Japanese)

[22] Tohara H, Saitoh E, Mays KA, Kuhlemeier K, Palmer JB. Three tests for predicting aspiration without videofluorography. Dysphagia 2003; 18: 126–134. 10.1007/s00455-002-0095-y 12825906

[23] Glantz SA, Slinker BK. Multicollinearity and What to Do About It In: Glantz SA, Slinker BK, editors. Primer of Applied Regression and Analysis of Variance. 2nd ed New York: McGraw-Hill Education; 2001 Pp. 185–240.

[24] Smithard DG, O'Neill PA, England RE, Park CL, Wyatt R, Martin DF, et al The natural history of dysphagia following a stroke. Dysphagia 1997; 12: 188–193. 9294937 10.1007/PL00009535

[25] Coates C, Bakheit AM. Dysphagia in Parkinson's disease. Eur Neurol 1997; 38: 49–52. 9252799 10.1159/000112902

[26] Good-Fratturelli MD, Curlee RF, Holle JL. Prevalence and nature of dysphagia in VA patients with COPD referred for videofluoroscopic swallow examination. J Commun Disord 2000; 33: 93–110. 10834828 10.1016/s0021-9924(99)00026-x

[27] Shiba N, Nochioka K, Miura M, Kohno H, Shimokawa H; on behalf of the CHART-2 Investigators. Trend of westernization of etiology and clinical characteristics of heart failure patients in Japan: First report from the CHART-2 Study. Circ J 2011; 75: 823–833. 21436596 10.1253/circj.cj-11-0135

[28] Alagiakrishnan K, Bhanji RA, Kurian M. Evaluation and management of oropharyngeal dysphagia in different types of dementia: a systematic review. Arch Gerontol Geriatr 2013; 56: 1–9. 10.1016/j.archger.2012.04.011 22608838

[29] Suh MK, Kim H, Na DL. Dysphagia in patients with dementia: Alzheimer versus vascular. Alzheimer Dis Assoc Disord 2009; 23: 178–184. 10.1097/WAD.0b013e318192a539 19474573

[30] Prince M, Bryce R, Albanese E, Wimo A, Ribeiro W, Ferri CP. The global prevalence of dementia: a systematic review and metaanalysis. Alzheimers Dement 2013; 9: 63–75. 10.1016/j.jalz.2012.11.007 23305823

[31] Zuccalà G, Cattel C, Manes-Gravina E, Di Niro MG, Cocchi A, Bernabei R. Left ventricular dysfunction: a clue to cognitive impairment in older patients with heart failure. J Neurol Neurosurg Psychiatry 1997; 63: 509–512. 9343133 10.1136/jnnp.63.4.509 PMC2169754

[32] Gruhn N, Larsen FS, Boesgaard S, Knudsen GM, Mortensen SA, Thomsen G, et al Cerebral blood flow in patients with chronic heart failure before and after heart transplantation. Stroke 2001; 32: 2530–2533. 11692012 10.1161/hs1101.098360

[33] Oh JE, Shin JW, Sohn EH, Jung JO, Jeong SH, Song HJ, et al Effect of cardiac function on cognition and brain structural changes in dementia. J Clin Neurol 2012; 8: 123–129. 10.3988/jcn.2012.8.2.123 22787496 PMC3391617

[34] Choi BR, Kim JS, Yang YJ, Park KM, Lee CW, Kim YH, et al Factors associated with decreased cerebral blood flow in congestive heart failure secondary to idiopathic dilated cardiomyopathy. Am J Cardiol 2006; 97: 1365–1369. 10.1016/j.amjcard.2005.11.059 16635612

[35] Mirza SS, de Bruijn RF, Koudstaal PJ, van den Meiracker AH, Franco OH, Hofman A, et al The N-terminal pro B-type natriuretic peptide, and risk of dementia and cognitive decline: a 10-year follow-up study in the general population. J Neurol Neurosurg Psychiatry 2015; 27. accessed March 9, 2016. 10.1136/jnnp-2014-309968 25918047

[36] Arora NS, Rochester DF. Respiratory muscle strength and maximal voluntary ventilation in undernourished patients. Am Rev Respir Dis 1982; 126: 5–8. 7091909 10.1164/arrd.1982.126.1.5

[37] Shizgal HM, Vasilevsky CA, Gardiner PF, Wang WZ, Tuitt DA, Brabant GV. Nutritional assessment and skeletal muscle function. Am J Clin Nutr 1986; 44: 761–771. 3098084 10.1093/ajcn/44.6.761

[38] Veldee MS, Peth LD. Can protein-calorie malnutrition cause dysphagia? Dysphagia 1992; 7: 86–101. 1572231 10.1007/BF02493439

[39] Hudson HM, Daubert CR, Mills RH. The interdependency of protein-energy malnutrition, aging, and dysphagia. Dysphagia 2000; 15: 31–38. 10.1007/s004559910007 10594256

[40] Mirzakhani H, Williams JN, Mello J, Joseph S, Meyer MJ, et al Muscle weakness predicts pharyngeal dysfunction and symptomatic aspiration in long-term ventilated patients. Anesthesiology 2013; 119: 389–397. 10.1097/ALN.0b013e31829373fe 23584384

[41] Wójkowska-Mach J, Gryglewska B, Romaniszyn D, Natkaniec J, Pobiega M, Adamski P, et al Age and other risk factors of pneumonia among residents of Polish long-term care facilities. Int J Infect Dis 2013; 17: e37–43, accessed March 9, 2016. 23041365 10.1016/j.ijid.2012.07.020

[42] Katsura H, Yamada K, Kida K. Outcome of repeated pulmonary aspiration in frail elderly. The Project Team for Aspiration Pneumonia. Nihon Ronen Igakkai Zasshi 1998; 35: 363–366. (in Japanese) 9711090 10.3143/geriatrics.35.363

[43] Hillel AD, Yorkston K, Miller RM. Using phonation time to estimate vital capacity in amyotrophic lateral sclerosis. Arch Phys Med Rehabil 1989; 70: 618–620. 2764692

